# Growth Retardation, General Hypotonia, and Loss of Acquired Neuromotor Skills in the Infants of Mothers With Cobalamin Deficiency and the Possible Role of Succinyl-CoA and Glycine in the Pathogenesis

**DOI:** 10.1097/MD.0000000000000584

**Published:** 2015-03-06

**Authors:** Zafer Bicakci

**Affiliations:** From the Department of Pediatric Hematology, Kafkas University Faculty of Medicine, Paşa Çayırı, Kars, Turkey.

## Abstract

Vitamin B_12_ (cobalamin, Cbl) deficiency can cause metabolic, hematological, and neurological abnormalities. Adequate levels of succinyl-coenzyme A (CoA) cannot be synthesized from methylmalonyl-CoA because of the decreased activity of the methylmalonyl-CoA mutase enzyme that uses Cbl as the cofactor. Succinyl-CoA synthesis deficiency leads to decreased heme synthesis and gluconeogenesis. The reason of growth retardation can be gluconeogenesis deficiency together with heme synthesis deficiency whereas the reason of the neurological abnormalities can be glycine increase in the tissue due to decreased heme synthesis. We present 7 infants diagnosed with severe nutritional Cbl deficiency and discuss the role of succinyl-CoA and glycine in the possible pathogenesis in this article. Patients brought to our clinic with a complaint of growth retardation and diagnosed with nutritional Cbl deficiency were included in the study. There were 5 females and 2 males. The mean age was 11 ± 2.30 (range 6–13) months. All patients had general muscular hypotonia and 4 had growth retardation. Neuromotor growth retardation was found in 4 of the children who had previously shown normal neuromotor development for age. The mean Cbl level was 83.8 ± 27.6 (45.6–114) pg/mL. The mean Cbl level of the mothers was 155 ± 56.6 (88–258) pg/mL. Six of the patients had anemia and 1 had thrombocytopenia. Mean corpuscular volume value was 91.5 ± 12.2 fL. Following treatment, the muscle tonus of the patients improved, the anemia and growth retardation decreased, and the lost neuromotor abilities were recovered. Severe nutritional Cbl deficiency is an important nutritional disease where complications can be prevented with early treatment. When evaluating the pathogenesis, it should be noted that nutritional Cbl deficiency is a succinyl-CoA synthesis deficiency.

## INTRODUCTION

Vitamin B_12_ (cobalamin, Cbl) is an essential vitamin. The most common reason of Cbl deficiency in infancy is being fed only with milk of a mother with Cbl deficiency.^1^ Nutritional Cbl deficiency usually starts in infants aged 6 to 12 months with vomiting, lethargy, tremors, abnormal movements, growth retardation, hypotonia, and loss of developmental skills. There may be increased methylmalonic acid and homocysteine in the blood and sometimes hyperglycinuria.^2–4^ Methylmalonic acidemia (MMA) results from deficiency of either methylmalonyl-CoA mutase (MCM) or defects in the production of adenosylcobalamin (AdoCbl). Deficiency of Cbl, a cofactor for MCM, also produces signs and symptoms consistent with MMA. Other biochemically closely related disorders are the Cbl defects, classified as forms A through G. Clinical signs and symptoms include failure to thrive, metabolic acidosis, persistent ketotic episodes, hypoglycemia, hypotonia, hyperammonemia, and neurologic symptoms. Increased methylmalonic acid, homocysteine, and glycine may be present in the blood and urine.^5^

Although hyperglycinemia and hyperglycinuria are frequently reported in MMA, only hyperglycinuria is reported in severe nutritional Cbl deficiency.^3–8^ Clinical signs and symptoms are similar in severe nutritional Cbl deficiency and MMA. Hyperglycinurina is seen in both cases due to the same enzymes/cofactors whereas the hyperglycinemia seen in MMA is also expected in severe nutritional Cbl deficiency. Glycine and succinyl-CoA are combined with a reaction catalyzed by δ-amino levulinic acid (ALA) synthase and δ-ALA is formed.^9^ The synthesis of succinyl-CoA from methylmalonyl-CoA decreases because of the level of MCM enzyme activity to (enzyme/AdoCbl) Cbl deficiency.^10,11^ The insufficient synthesis of succinyl-CoA cannot fully compensate for the sufficient synthesis of glycine and can lead to insufficient heme synthesis. Therefore, the glycine that cannot combine and react with succinyl-CoA and that possibly increases above the elimination capacity can deposit in the tissues.

Cbl plays an essential role in mitochondrial energy production (gluconeogenesis) and cellular functions.^12^ Cbl deficiency causes a severe growth retardation and various metabolic disorders in animals.^13^ Growth retardation is commonly reported in nutritional Cbl deficiency.^14–16^ However, there is little emphasis on the reason(s). Iron or Cbl stores are consumed approximately in 6 months, with nutritional anemia possibly developing afterward, in infants who have mothers with iron and Cbl deficiency and are only fed breast milk.^17–19^ Although rapid growth increases the need for iron and causes iron deficiency anemia in infants, Cbl deficiency in the same period can slow the growth rate and cause growth retardation and megaloblastic anemia.^14–17^ With regard to the contrasting effect on growth of these 2 causes of anemia, the insufficient energy (glucose) production as a result of the gluconeogenesis deficiency could be a factor increasing growth retardation in addition to the effect of the Cbl deficiency anemia.^12^

The increase of glycine in tissues such as the central nervous system (CNS) due to insufficiency of succinyl-CoA may lead to the emergence of abnormal nervous system findings.^18^ Although there are a large number of publications on abnormal nervous system findings in nutritional Cbl deficiency, only a few publications address the role of glycine in the pathogenesis of these abnormalities.^2,20^

We present 7 infants diagnosed with severe nutritional Cbl deficiency who suffered from growth retardation and general muscle hypotonia and had lost acquired neuromotor skills, and discuss the role of succinyl-CoA and glycine in the possible pathogenesis in this article.

## PATIENTS AND METHODS

Kars is a province in the Northeastern Anatolia region of Turkey and is about 1800 to 2000 m above the sea level. It is relatively undeveloped socially and financially. The main source of income is agriculture and livestock. The patients had come from various villages of Kars. This study was designed as a retrospective trial. For that reason, we took a permission in order to scan the patients’ files from our institutional committee. Patients who were brought to our clinic for growth retardation between 2006 and 2013 and were diagnosed with nutritional Cbl deficiency were included in the study. The age ranged from 6 to 13 months. All patients were evaluated with a full history, and physical and neurological examination. The Cbl, folic acid, complete blood count, peripheral blood smear, serum iron (SI), serum iron-binding capacity (SIBC), ferritin, total bilirubin (TB) and direct bilirubin (DB), lactate dehydrogenase (LDH) levels of the patients, and the Cbl and folic acid levels of the mothers were studied. Megaloblastic anemia related to Cbl deficiency was diagnosed with a combination of clinical and laboratory findings, including low Cbl level, neutrophil hypersegmentation, and increased mean corpuscular volume (MCV) (macrocytosis). Growth retardation was described as weight under 3% for the patient's age. Neuromotor growth retardation was described as a general slowing of mental and physical activity and a fundamental failure to reach the developmental stages. The loss of acquired neuromotor skills was described as the inability to hold the head straight, inability to sit without support, inability to crawl, and inability to walk in children who had previously shown normal neuromotor development for their age. Cbl treatment was administered intramuscularly at a dose of 100 μg/d for 1 week, followed by every 2 days for 1 week, 3 days a week for 1 week, once a week for 1 week, and then 1000 μg once a month for 6 months.^21^ Iron and folic acid were not administered. We did not check MMA and homocysteine levels in the patients and therefore confirmed our diagnosis by comparing the laboratory values at the time of diagnosis and 1 year later (after treatment).

## RESULTS

There were 5 females and 2 males. The mean age was 11 ± 2.30 (range 6–13) months. All patients were fed breast milk. The families of the patients were from a low socioeconomic level. The mothers of all patients had an inadequate intake of animal products. Length, head circumference, and body weight were under 3% in 4 patients and between 3% and 10% in 3 patients according to age. All patients had loss of appetite, pallor, and general muscular hypotonia. Four had loss of deep tendon reflexes. Four children had previously shown normal neuromotor development for age but were now unable to hold the head straight, sit without support, crawl, or walk (Table 1). The mean Cbl level was 83.8 ± 27.6 (45.6–114) pg/mL. The mean Cbl level of the mothers was 155 ± 56.6 (88–258) pg/mL. Six of the patients had anemia and 1 had thrombocytopenia. The patients’ mean serum ferritin, transferrin saturation (TS), and LDH levels were high, the mean SIBC was low, and the mean SI level was normal. MCV value was 91.5 ± 12.2 fL (Tables 2 and 3). Macrocytosis and anisocytosis were present in the peripheral smear of all patients. Proteinuria was not present in any patient. Convulsions or tremors were not observed in any patient before or after the treatment. Treatment led to improved general muscle tonus, decreased anemia and growth retardation, and recovery of the neuromotor abilities that had been lost. The patients’ hemoglobin (HGB), hematocrit, erythrocyte, and thrombocyte levels had increased and the MCV, MCH, and RDW levels decreased whereas the white blood cells and mean corpuscular hemoglobin concentration had not changed much with treatment 1 year after the diagnosis. The mean ferritin, SI, TS, LDH, folic acid, and TB and DB levels had decreased whereas the SIBC and Cbl levels had increased with treatment 1 year after the diagnosis (Tables 4 and 5).

**TABLE 1:**
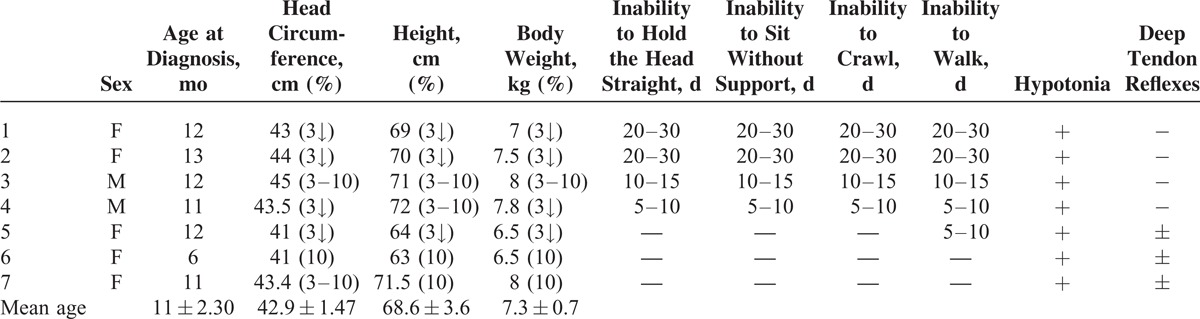
Patient Age of Diagnosis and Some Growth and Neuromotor Capacity-Related Findings

**TABLE 2:**
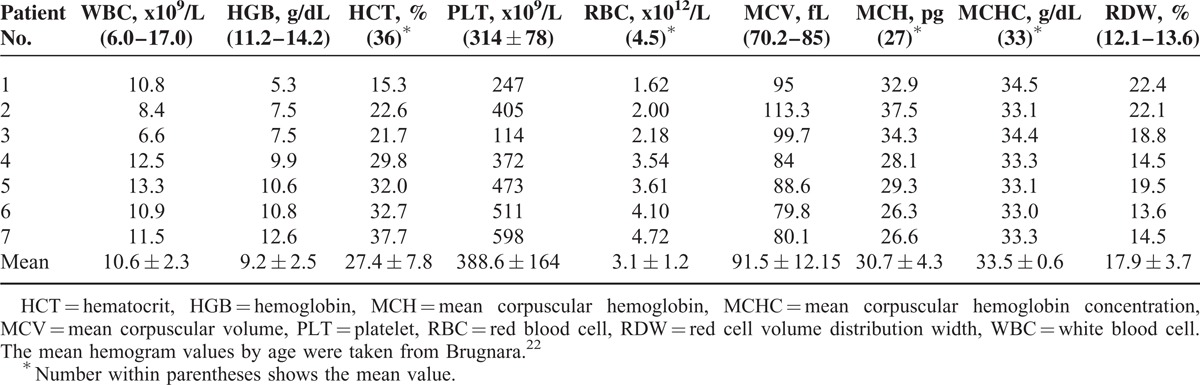
Pretreatment Hemogram Findings of the Patients

**TABLE 3:**
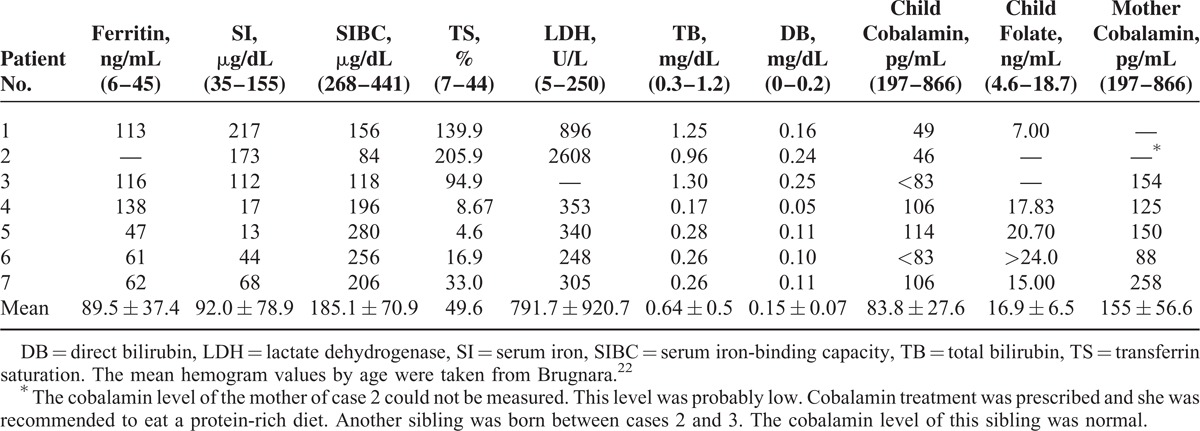
Some Pretreatment Laboratory Findings of the Patients and Their Mothers

**TABLE 4:**
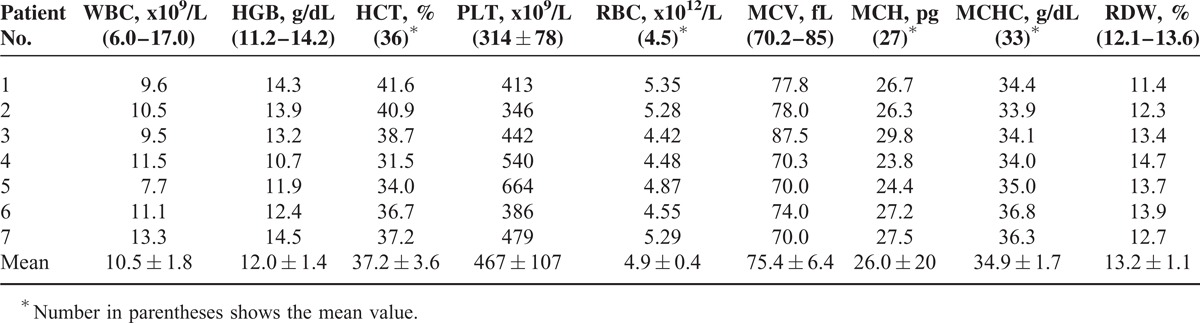
Posttreatment Hemogram Findings of the Patients

**TABLE 5:**
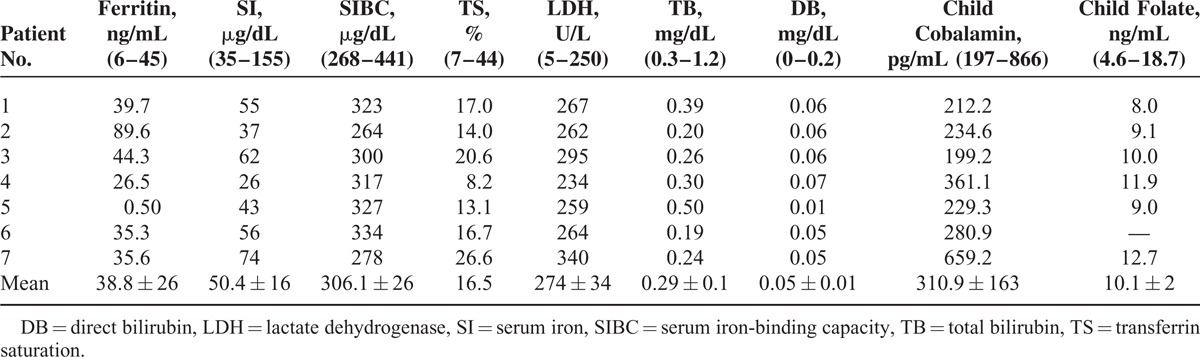
Some Posttreatment Laboratory Findings of the Patients

## DISCUSSION

Nutritional Cbl deficiency is usually seen in infants fed only breast milk of mothers who are vegetarian or who have not yet been diagnosed with pernicious anemia in developed countries. The most common cases in developing countries are nursing infants of mothers who eat a diet poor in protein. Nutritional Cbl deficiency is the primary cause of megaloblastic anemia in lower socioeconomic groups. Cbl deficiency is most commonly seen in developing parts of the world such as India, Africa, Mexico, and Middle and South America countries.^1^ Although adults fed with a diet poor in Cbl can use their endogenous Cbl, infants fed a diet lacking Cbl can become symptomatic usually within 6 to 12 months after birth due to their limited stores.^23^ Our patients were aged between 6 and 13 months. This age range is consistent with the vitamin B_12_ stores depletion age range of infants reported in the literature.

### Metabolic Abnormalities

#### Succinyl-CoA Homeostasis

Succinyl-CoA is mainly synthesized from 2 sources. It is synthesized from α-ketoglutarate with α-ketoglutarate dehydrogenase through decarboxylation and CoA is added during this process. It is also synthesized from propionyl-CoA that is formed as a result of the catabolism of single-chain fatty acids and some branched-chain amino acids and cholesterol. Succinyl-CoA is a key intermediate metabolite of the citric acid cycle and can be easily included in this cycle. Succinyl-CoA is metabolized in 2 ways. It is converted to succinate with succinyl-CoA synthesis (succinate thiokinase) and CoA is hydrolyzed. Succinyl-CoA and glycine are also combined by δ-ALA synthase to form δ-ALA for the synthesis of porphyrin (heme), ensuring the homeostasis of succinyl-CoA.^9,24,25^

#### Gluconeogenesis

Gluconeogenesis is the process of the synthesis of glycogen or glucose from noncarbohydrate precursors. The major substrates are the glycogenic amino acids, lactate, glycerol, and propionate. The liver and kidneys are the major glucogenic tissues, but the small intestines can also be a source of glucose in the fasting state. Glycogenic amino acids are converted to pyruvate or citric acid cycle intermediate metabolites after transamination or deamination. Gluconeogenesis reactions are therefore responsible for the conversion of both lactate and glycogenic amino acids into glucose or glycogen. Propionic acid is the major precursor of glucose in ruminants. Propionic acid is a relatively minor precursor of glucose in humans. Propionic acid is an intermediate metabolite of isoleucine, valine, threonine, methionine, single-chain fatty acids, and cholesterol catabolism. It is also synthesized by intestinal bacteria. Propionic acid enters gluconeogenesis with the citric acid cycle. Propionyl-CoA is formed after the esterification of propionic acid and CoA by acyl-CoA synthase. Propionyl-CoA becomes carboxylated with a reaction catalyzed by biotin-dependent propionyl-CoA to create d-methylmalonyl-CoA. d-methylmalonyl-CoA is then converted to l-methylmalonyl-CoA by methylmalonyl-CoA racemase. l-methylmalonyl-CoA is converted into succinyl-CoA molecules with the MCM enzyme for which the cofactor is AdoCbl.^25^

#### Cbl Metabolism Disorders

The Cbl in food enters cells as hydroxycobalamin. It is processed in the lysosomes and metabolized into the methionine synthase cofactor methylcobalamin or the methlmalonyl-CoA mutase cofactor AdoCbl. Patients with a Cbl metabolism disorder can develop homocystinuria alone, methylmalonic aciduria alone, or homocystinuria and methylmalonic aciduria in combination instead of a metabolic block. At least 8 different defects in the intracellular metabolism of Cbl have been defined. These are *cbl*A to *cbl*H; *cbl*A, *cbl*H, and *cbl*B cause MMA. The *cbl*C, *cbl*D, and *cbl*F defects cause homocystinuria in addition to MMA as both AdoCbl and methylcobalamin syntheses are disturbed. The *cbl*E and *cbl*G defects only involve methylcobalamin synthesis and cause homocystinuria without MMA. Megaloblastic anemia is usually present with these defects. The serum Cbl and folic acid levels are normal.^26^

#### Methionine Synthase Deficiency

A decrease in this enzyme's activity can frequently cause feeding disorders, lethargy, developmental delay, and megaloblastic anemia in the patients. Neurological problems and macrocytic anemia have been reported in adult patients. The serum Cbl and folic acid levels are normal.^27^

#### Methylmalonic Acidemia

There are many clinical pictures from very severely affected newborns to asymptomatic adults, independent of the nature of the enzymatic defect or the biochemical abnormality. Isolated MMA develops as a result of mutations in genes related to MMA, the MUT locus that encodes the MCM apoenzyme, or AdoCbl that is the cofactor of this enzyme. Two types of MCM deficiency are recognized. There is no enzyme activity in the “MUT^0^” form. There is a structural defect in the enzyme but some residual enzyme activity in the “MUT^−^” form.^28^ The deficiency of MCM or its cofactor AdoCbl leads to MMA. Disorder in MCM activity leads to methylmalonyl-CoA accumulation and this in turn causes an increase in propionyl-CoA and its metabolites by suppressing propionyl-CoA carboxylase, whereas both conditions cause methylmalonic and propionic acid accumulation in the blood and excretion through the urine. Laboratory findings are ketosis, acidosis, anemia, neutropenia, thrombocytopenia, hyperglycinemia, hyperammonemia, hypoglycemia, and excess methylmalonic acid in all body fluids. Propionic acid and its metabolites 3-hydroxypropionate and methylcitrate are also present in urine.^29^ The synthesis of succinyl-CoA from methylmalonyl-CoA decreases because of the level of MCM enzyme activity to (enzyme/AdoCbl) in nutritional Cbl deficiency.^10,11^ The inadequate synthesis of succinyl-CoA cannot fully meet the adequate synthesis of glycine and may result in inadequate heme synthesis. We believe that the glycine that cannot combine and react with succinyl-CoA probably increases over the elimination capacity (*“*cleavage” enzyme complex, loss with urine, heme synthesis, etc.) and deposits in the tissues.

#### Growth Retardation

Growth is regulated by genetic factors as well as hormones, tissue-specific growth factors, nutrition, and the interaction of many other internal and external environment factors. Normal growth can only be achieved by adequate nutrition. More than 10% of the energy received from food is spent for growth during rapid growth periods. Protein intake is also important for normal cell growth. A child organism where the structural and energy-providing materials are not adequate first tries to survive by stopping growth and development.^30^ Growth retardation (as reflected in the decreased growth proportional with the head circumference, height, and body weight) is therefore observed as the first and striking finding in MMA, an energy production disorder.^6,29^

Growth retardation is reported as the most striking clinical finding in infants with nutritional Cbl deficiency.^14–16^ A study has reported plasma methylmalonic acid and homocysteine levels 2 to 8 times of control values that were inversely associated with the significantly low Cbl levels in 41 infants on a macrobiotic diet. Although there was no neurological or hematological deficiency, these children were reported to show psychomotor development disorders and growth retardation when compared with the control group children who received a balanced diet.^31^ The mean values of all anthropometric parameters were low in infants aged 4 to 18 months of mothers on a macrobiotic diet in another study. Weight and arm circumference growth rate have been found to be independent of the protein content of the macrobiotic diet and the energy intake. Growth has been reported to be positively associated with the protein content of the diet but not with energy intake.^32^ The presence of severe growth retardation (proportionate growth retardation consistent with 3rd to 10th percentiles for the head circumference, height, and body weight) in almost all the patients in our study, improvement after treatment, and catching up with normal growth and development values within the first year of treatment can be explained with the effects of Cbl at the molecular level.

Insufficient synthesis of heme-containing proteins, primarily HGB, and insufficient gluconeogenesis may cause growth retardation because of insufficient succinyl-CoA production in Cbl deficiency. Heme-containing proteins (HGB, myoglobin, cytochrome enzymes, catalase, and tryptophan pyrolase) are synthesized in all cells except mature erythrocytes. Most of the heme synthesis in the body takes place in the bone marrow erythroid cells and most of the remaining part is in the liver hepatocytes and other heme-synthesizing cells.^9^ Inadequate heme/HGB synthesis (the effect of chronic anemia) leads to inadequate oxygenation (hypoxia), which, in turn, causes growth retardation with inadequate function in many tissues. The severe growth retardation observed in Cbl deficiency cannot only be explained by chronic anemia. Iron-deficiency anemia usually occurs as a result of rapid growth in infants fed only breast milk, and usually does not cause severe growth retardation. However, the cause of severe Cbl deficiency is nutritional deficiency and not rapid growth. Megaloblastic anemia and severe growth retardation are often observed as a result of severe Cbl deficiency.^14–16^ We therefore believe that the reason of the growth retardation in Cbl deficiency could be other factors, in addition to the anemia, that cause growth retardation, such as gluconeogenesis deficiency. Cbl deficiency leads to decreased/inadequate succinyl-CoA synthesis. This decrease leads to less energy (glucose) production by the citric acid cycle.^12^ The reason of the growth retardation could be gluconeogenesis deficiency together with heme synthesis deficiency. The presence of severe growth retardation and hypoglycemia in MMA, and the positive correlation of growth with the protein (and Cbl) content of the diet but not with energy intake may be further proof of a role for gluconeogenesis deficiency.^6,32,33^

### Hematological Abnormalities

#### Ineffective Hematopoiesis

A form of methionin synthase uses *N*^5^-methyltetrahydrofolate as the methyl (CH_3_) donor in common bacteria. Another form of the enzyme found in some bacteria and mammalians uses *N*^5^-methyltetrahydrofolate but first transfers the CH_3_ group to Cbl as the CH_3_ donor to produce methylcobalamin during methionine production. Methylcobalamin receives the CH_3_ groups from *N*^5^-methyltetrahydrofolate and this is the only reaction that uses *N*^5^-methyltetrahydrofolate in mammalians. The reaction that converts the *N*^5^,*N*^10^-methylene form into the *N*^5^-methyltetrahydrofolate form (with *N*^5^,*N*^10^-methylenetetrahydrofolate reductase) is irreversible. The metabolic folates therefore become trapped in the *N*^5^-methyl form if the coenzyme Cbl is not available for methylcobalamin synthesis. Megaloblastic anemia develops for the same reasons in folate deficiency where all forms of tetrahydrofolate are decreased.^34^

Erythroid precursors have increased RNA content but normal DNA content. The amount of RNA per unit of erythroid precursor DNA is increased and the cell is therefore larger than a normal cell at the same level of maturation. The nuclear chromatin of these cells appears looser than normal on the peripheral smear. This gives the megaloblasts their characteristic appearance. The maturation of the nucleus and cytoplasm is asynchronous and the nucleus is less mature than the cytoplasm.^21^ The problem in the development of erythroid precursors is the result of the direct consumption of *N*^5^,*N*^10^-methylenetetrahydrofolate. Thymidylate synthase is used as a C1-donor for the synthesis of thymidine nucleotides in the biosynthesis of the folate cofactor *N*^5^,*N*^10^-methylenetetrahydrofolate during DNA biosynthesis. A methylene group (–CH_2_–) bound to tetrahydrofolate is used by the thymidylate synthase enzyme to convert the uracil-type pyrimidine base in RNA (deoxyuridine monophosphate) to a thymine-type base in DNA (deoxythymidine monophosphate). Cbl deficiency that causes pseudofolate and folate deficiency decreases deoxythymidine triphosphate synthesis (as *N*^5^,*N*^10^-methylenetetrahydrofolate is consumed) and DNA with high uracil content forms after misinsertion of uracil into DNA during replication.^35,36^

Decreased Cbl (pseudofolate)/folate leads to decreased purine and pyrimidine biosynthesis and therefore decreased DNA biosynthesis and cell division. This is reflected in the decreased number of erythrocytes, causing anemia in the most direct manner.^36^ Treatment makes the coenzyme Cbl available for methylcobalamin synthesis and the metabolic folates are no longer trapped in their *N*^5^-methyl form. They are converted into tetrahydrofolate, restarting the production of *N*^5^,*N*^10^-methylenetetrahydrofolate. Thymidylate synthase uses the folate cofactor *N*^5^,*N*^10^-methylenetetrahydrofolate for the resynthesis of thymidine nucleotides (DNA synthesis). Cell division starts to increase again and this may cause the megaloblastic changes in the bone marrow to recover.

#### Inadequate Synthesis of Heme

However, the disturbed de novo synthesis of thymidine nucleotides may partially explain the megaloblastic anemia. Observations in animal models and nonanemic patients with Cbl deficiency have shown that thymidylate synthase deficiency by itself is not enough to cause anemia. There may be other contributing factors.^37^ One of these other factors may be the inadequate synthesis of the succinyl-CoA required for heme synthesis.

Serum bilirubin, iron, TS, and LDH levels can increase because of ineffective hematopoiesis and in correlation with the severity of anemia.^21^ Although our patient number is inadequate (Table 3), ferritin and LDH levels were high and showed an inverse correlation with the severity of the anemia. However, the mean SIBC had decreased and the mean TS increased. The mean SI was normal. The maturation of megaloblasts and the 50% decrease of SI and LDH levels 24 hours after Cbl treatment can be associated with rapid iron use.^21,38^ Production of succinyl-CoA increases with the activation of the MCM enzyme as a result of Cbl treatment. We believe the increased succinyl-CoA production could increase heme synthesis and thus iron use and rapidly reduce SI levels.

A “positive therapeutic test” criteria for Cbl deficiency diagnosis is a 50% decrease in SI and LDH levels.^21^ However, this criterion is not used often for folic acid deficiency. One of the differences between these disorders may be the rapid decrease in SI after treatment as it is used for production. The serum ferritin level may increase in Cbl deficiency, because of both ineffective erythropoiesis and inadequate heme synthesis. The mean serum ferritin level a year after the diagnosis of Cbl deficiency in our patients (38.8 ± 26 after treatment) was less than half of the level at diagnosis (89.5 ± 37.4 before treatment). This decrease in the serum ferritin level may indicate that the iron required for heme synthesis is not utilized adequately.

### Neurological Abnormalities

Cbl is primarily required for hematopoiesis and nervous system development. Cbl deficiency in adults can be seen as megaloblastic anemia, polyneuropathy, subacute combined neurodegeneration of the spinal cord, dementia, or depression. Although a clinical disorder due to Cbl deficiency develops slowly over months or years in the mature (adult) nervous system, significant clinical problems can develop within a couple of weeks in infants with rapid cerebral growth and development. The most commonly seen symptoms are growth retardation, hypotonia, hyperirritability or lethargy, neuromotor delay or retardation, epilepsy, and abnormal movements.^39^

The cerebrospinal fluid (CSF) methylmalonic acid concentration had increased (the mean level was 600 times that of controls) in a disproportionate manner to the serum values in 3 patients with neuropsychiatric syndrome. However, the potential benefit of CSF metabolite levels in the diagnosis of Cbl deficiency has not been determined.^40,41^ The level of methylmalonic acid increases at CSF in the deficiency of Cbl. Because of this reason, the level of glycine is expected to increase in the CNS.

Urinary concentrations of methylmalonic acid and homocysteine are typically high in Cbl deficiency. Hyperglycinuria is occasionally present. There are 2 reports on infants with Cbl deficiency showing abnormal movements after the start of the treatment. Methylmalonic acid and homocysteine were reported to disappear quickly after the start of treatment in 2 infants where abnormal movements were present but the hyperglycinuria that was present at the beginning of the abnormal movements continued.^2^

The fact that glycine does not always increase in the blood and urine such as methylmalonic acid and homocysteine can be related to the cleavage enzyme complex or other metabolic pathways that can catabolize/eliminate glycine easily. Although glycine cannot pass the blood–brain barrier easily, the glycine increase in the CSF in nonketotic hyperglycinemia is almost 2 to 4 times the increase in the serum.^42,43^ This indicates the possible presence of other catabolism/elimination routes outside the CNS other than the cleavage enzyme complex. The increase of glycine primarily in the nervous system due to Cbl deficiency, even if at the cell and tissue level and not in the blood, can explain the abnormal clinical findings stemming from the nervous system.

Glycine is an important excitatory neurotransmitter released from the inhibitory interneurons in the spinal cord and brain stem, and is an agonist of the glycine receptors. A large part of the interneurons in the spinal cord gray matter consists of glycinergic neurons whereas a smaller part is Gamma Amino Butyric Acid (GABA)ergic neurons. Glycine is effective on 2 different receptors in the CNS. The glycine receptor consists of α, β, and other subunits; it has a chloride channel in the middle such as the GABA receptor. The receptors clamped to the chloride channel are called glycine_A_ receptors. Strychnine antagonizes the effect of glycine not at the glycine receptor level but at the level of the chloride channel. Some glycine-binding sites on the glutamate *N*-methyl-d-aspartate (NMDA) receptors that are chloride channel locked cannot be blocked with strychnine. These are called glycine_B_ receptors.^44–46^

Inhibitor glycine receptors (glycine_A_) are mainly found in the spinal cord, brainstem, and retina.^45^ Stimulation of the glycine inhibitor receptor causes lethargy and respiratory depression.^47,48^ The most common neurological findings in infants with severe Cbl deficiency have been reported as hypotonia, growth retardation, and the loss of acquired neuromotor skills.^20,39,49^ All our patients had hypotonia and our study was consistent with the literature in this regard. Although we failed to show glycine elevation in our study, the presence of hypotonia before the treatment, its improvement with treatment, and the lack of any sequel indicates the presence of a functional inhibitory neurotransmitter such as glycine. The reason for the neurological findings could be the inhibitory effect of the increasing glycine level on glycinergic neurons (stimulating glycine_A_ receptors), although at the cellular level, before treatment and the disappearance of the inhibitory effect due to the decreased glycine level after the treatment. In addition, a large part of the interneurons in the spinal cord gray matter consists of glycinergic neurons that are especially responsible for the glycinergic inhibitory neurotransmission (with α3β glycine receptors) in the dorsal horn of the spinal cord.^45^ The subacute combined neurodegeneration in the posterolateral spinal cord of adult patients with Cbl deficiency indicates that the glycinergic neurons that we believe glycine causes hypotonia through (stimulating glycine_A_ receptors) and the neurons where subacute combined neurodegeneration are observed could be similar neurons.

Glycine is also the coagonist of glutamate in glutamatergic NMDA receptors in the brain (allosteric) and therefore has both excitatory and inhibitory functions. Glycine transporters (GlyT) eliminate glycine from the synaptic cleft of the glycinergic inhibitory neurons in synapses containing glycine receptors in the CNS and this process maintains the low glycine extracellular concentration necessary to saturate the glycine site over the glutaminergic NMDA receptors. The human GlyT1 transporter is Na^+^/Cl^−^ dependent and is expressed in the glia. GlyT2 transporter is expressed in the neurons and recovers the released glycine from the synaptic gap. Glycine concentration in inhibitory neurons is higher than the glycine concentration in other parts of the CNS (2–3 times difference). Glycinergic inhibitory neurotransmission ends with recovery of GlyT2 into neuronal terminals and GlyT1 into glia. GlyT1 also prevents the saturation of the glycine-binding site over the NMDA receptors.^42^ The stimulation of NMDA receptors with glycine leads to hyperirritability and an inability to perform voluntary movements.^47,48^ The excessive stimulation of the NMDA receptor usually also contributes to the neurological damage related to nonketotic hyperglycinemia.^50^ Abnormal or severe involuntary movements accompanied by tremor and myoclonus, twitching, and chorea affecting particularly the tongue, face, pharynx, and legs can be seen in about half of the infants with Cbl deficiency. These movements may improve before or a few days after the start of the treatment. The duration of the movements change between 10 and 30 days.^2,51,52^ All our patients had neuromotor growth retardation and most of them had lost their acquired neuromotor skills. We believe that the presence of neuromotor development delay and acquired neuromotor ability loss before the treatment and their improvement with treatment, generally without any sequelae, indicates a role for a functional inhibitory neurotransmitter such as glycine. The stimulation of glycine_B_ receptors by glycine may be responsible for the abnormal movements before treatment as the increased glycine at the cellular level leads to an inability of GlyT1 to saturate the glycine-binding area through the NMDA receptor. Normal functioning of glycinergic neurons is necessary to provide a balance between the excitatory and inhibitory activity in the integrative centers in the spinal cord that regulate skeletal muscle contractions. We believe that the reason of the abnormal movements or convulsions is the temporary imbalance brought by the rearrangement of glycine_A_/glycine_B_ receptors and GlyT1/GlyT2 together with the decrease of glycine at the cellular level. Glycine is the coagonist of glutamate in glutamatergic NMDA receptors in the brain and may therefore have both excitatory and inhibitory functions and be the cause of abnormal movements or convulsions.

The folate form (*N*^5^,*N*^10^-methylenetetrahydrofolate) used for thymidylate synthesis can alternatively also be channeled into the “methylation cycle.” This ensures adequate *S*-adenosylmethionine for the cell at all times. *S*-adenosylmethionine is a methionin-activated form that acts as a CH_3_ donor for many methyltransferase enzymes. These enzymes add a CH_3_ group to a wide range of substrates such as lipids, hormones, proteins, and DNA. One of these important methylations concerns myelin basic protein. This protein constitutes a third of the myelin cover of nerves, providing insulation. The methylation cycle is interrupted in Cbl deficiency and causes nerve demyelinization.^35^

Although at the hypothesis level, our study has demonstrated that Cbl deficiency is a substrate/succinyl-CoA deficiency. Succinyl-CoA deficiency causes 2 important biochemical pathways to function inadequately. The result is heme synthesis deficiency and gluconeogenesis deficiency. Heme synthesis deficiency together with gluconeogenesis deficiency (energy production [glucose] deficiency) can be a reason of growth retardation. In addition, the cause of the neurological abnormalities can be increased glycine in tissues due to the heme synthesis (succinyl-CoA) deficiency.^10–16^

Despite the limitations of the inadequate number of patients and the inability to measure methylmalonic acid, homocysteine, and glycine levels in our study, we think that it is important to systematically summarize the reasons of growth retardation, neuromotor skill loss, and hyperglycinemia even at the hypothesis level in light of essential information and believe that our study will shed light on future studies.

In conclusion, nutritional Cbl deficiency may be observed in various clinical forms from asymptomatic disease to severe disease according to the Cbl level. Severe Cbl deficiency is an important nutritional disorder and the complications can be prevented with early treatment. When evaluating the pathogenesis of the disorder, it should be noted that nutritional Cbl deficiency is a succinyl-CoA synthesis deficiency.
